# Whole genome sequencing of matched tumor, adjacent non-tumor tissues and corresponding normal blood samples of hepatocellular carcinoma patients revealed dynamic changes of the mutations profiles during hepatocarcinogenesis

**DOI:** 10.18632/oncotarget.15428

**Published:** 2017-02-17

**Authors:** Ruifang Mao, Jie Liu, Guanfeng Liu, Shanshan Jin, Qingzhong Xue, Liang Ma, Yan Fu, Na Zhao, Jinliang Xing, Lanjuan Li, Yunqing Qiu, Biaoyang Lin

**Affiliations:** ^1^ Collaborative Innovation Center for Diagnosis and Treatment of Infectious Diseases, The First Affiliated Hospital, School of Medicine, Zhejiang University, Hangzhou, China; ^2^ Systems Biology Division, Zhejiang-California International Nanosystems Institute (ZCNI), Zhejiang University, Hangzhou, Zhejiang Province, P.R. China; ^3^ Departmant of Agronomy, College of Agriculture and Biotechnology, Zhejiang University, Hangzhou, P. R. China; ^4^ College of Animal Sciences, Zhejiang University, Hangzhou, P. R. China; ^5^ State Key Laboratory of Cancer Biology and Experimental Teaching Center of Basic Medicine, Fourth Military Medical University, Xi'an, China; ^6^ The First Affiliated Hospital, School of Medicine, Zhejiang University, Hangzhou, China; ^7^ Departmant of Urology, University of Washington, Seattle, WA, USA

**Keywords:** hepatocellular carcinoma (HCC), whole genome sequencing, next-generation sequencing, mutations, hepatitis B virus (HBV)

## Abstract

Hepatocellular carcinoma (HCC) has become the third most deadly disease worldwide and HBV is the major factor in Asia and Africa. We conducted 9 WGS (whole genome sequencing) analyses for matched samples of tumor, adjacent non-tumor tissues and normal blood samples of HCC patients from three HBV positive patients. We then validated the mutations identified in a larger cohort of 177 HCC patients. We found that the number of the unique somatic mutations (average of 59,136) in tumor samples is significantly less than that in adjacent non-tumor tissues (average 83, 633). We discovered that the TP53 R249S mutation occurred in 7.7% of the HCC patients, and it was significantly associated with poor diagnosis. In addition, we found that the L104P mutation in the VCX gene (Variable charge, X-linked) was absent in white blood cell samples, but present at 11.1% frequency in the adjacent tissues and increased to 14.6% in HCC tissues, suggesting that this mutation might be a tumor driver gene driving HCC carcinogenesis. Finally, we identified a TK1-RNU7 fusion, which would result in a deletion of 103 amino acids from its C-terminal. The frequencies of this fusion event decreased from the adjacent tissues (29.2%) to the tumors (16.7%), suggesting that a truncated thymidine Kinase1 (TK1) caused by the fusion event might be deleterious and be selected against during tumor progression. The three-way comparisons allow the identification of potential driver mutations of carcinogenesis. Furthermore, our dataset provides the research community a valuable dataset for identifying dynamic changes of mutation profiles and driver mutations for HCC.

## BACKGROUND

Hepatocellular carcinoma (HCC) is the most common primary liver malignancy with an estimated 600, 000 newly diagnosed cases every year worldwide [1, 2]. It is one of the most common virus- associated cancers known to be highly refractory to treatment and represents one of the leading causes of cancer death worldwide [1, 2]. The common etiology factors for HCC include chronic infections with hepatitis B (HBV) or hepatitis C (HCV) viruses, and exposure to dietary aflatoxin B1 (AFB1), alcohol consumption. Among them, hepatitis B virus (HBV) infection is the major cause of HCC in China and remains the major etiological factor in the HBV epidemic regions of China, South Korea, Southeast Asia, and sub-Saharan Africa [3]. Although liver cancer patients can be treated by surgery including liver transplantation, they have a low 5-year survival rate and a high recurrence rate [4–6].

Like other cancers, HCC can be considered as an acquired genetic disorder associated with an accumulation of somatic genetic alterations in hepatocytes [7]. During hepatocarcinogenesis, gross genomic alterations occur, which include chromosomal deletion or amplification, CpG methylation, DNA hypomethylation, DNA rearrangements [8, 9]. Previous studies of the pathogenesis of HCC have revealed genetic alterations and molecular characteristics in HBV-mediated HCC such as mutations in beta catenin (CTNNB1), CDKN2A, TP53 and AXIN1 [10–13], amplification of CCND1/FGF19 and MYC, HBV integration in TERT and MLL4 [14–16].

The emergence and rapid progress of next-generation sequencing (NGS) have enabled comprehensive characterization of cancers, including HCC [10, 17, 18]. Recently, a number of pioneering studies have published on whole genome or exome sequencing of HCC and refined our understanding of the mutational landscape and related signaling pathways involved in hepatocarcinogenesis [10, 11, 19–21]. However, instead of using true normal tissues (such as blood white cells) as the control for genetic background, most previous genomic analyses of HCC used so called normal adjacent tissue, which might already harbor precursor mutations. Such analysis might miss preneoplastic lesions in the adjacent tissues, some of which are potential driver mutations for liver carcinogenesis. The assumption of adjacent tissues as normal tissues might not be held as they might already harbor early genomic aberrations [22, 23]. In a previous systematic analysis of HBV integration in HCCs, we showed that HBV integration events exist in the tumor adjacent tissues and that a higher number of HBV integration events were found in the tumor adjacent tissues than in the HCC tissues, suggesting a clonal expansion process during HCC development [19]. We hypothesize that, similar to the HBV-integration events, HCC related mutations exist in the HCC adjacent tissues, and by a three way comparison of HCC tumors, tumor adjacent tissues and normal blood samples, we can identify early mutations in the tumor adjacent tissues, and better understand the dynamic changes of the mutations profiles during hepatocarcinogenesis. Hepatocellular carcinoma, one of the most common virus-associated cancers, is the third most frequent cause of cancer-related death worldwide and proved to be highly refractory to treatment. Hepatitis B virus (HBV) infection causes the majority of HCC cases.

To overcome the shortcoming in the previous analysis, we conducted a genome-wide screening of tumor, adjacent non-tumor tissue and normal white blood cells to gain insight into the molecular basis of tumor initiation and progression. We first conducted whole genome sequencing of matched samples of tumor, adjacent non-tumor tissues and normal blood samples of HCC patients. We then validated the mutations identified in a larger cohort of HCC patients by Sequenom MassARRAY platform. we found that the TP53 (R249S) mutation was found exclusively in tumor tissues occurring in 7.7% of the HCC patients (Supplementary Table 8). Furthermore, a survival analysis of the HCC patients with or without the TP53 (R249S) mutation showed that the HCC patients with the TP53 R249S mutation have significantly poor survival compared with those with the wild type P53 alleles. We found that the L104P mutation in the VCX gene (Variable charge, X-linked) was detected with increasing frequencies from normal, to adjacent tissues and then to tumor tissues; with frequencies of 14.6% in HCC, 11.1% in adjacent tissues, and absent (0%) in white blood cell samples.

## RESULTS

### Whole genome sequencing of matched tumor, non-tumor (somatic) and blood white cell (germline) samples of HCC patients

Using the Complete Genomics Inc.'s unchained combinatorial probe anchor ligation (cPAL) chemistry on arrays of self-assembling DNA nanoballs (DNBs) [24], we conducted whole genome sequencing of matched samples of tumor, adjacent non-tumor tissues and normal blood samples of HCC patients from three HBV positive patients, resulting in a total of nine whole genome sequences. We obtained a mean gross mapping reads of 182.37 Gb, 257.30 Gb, and 191.73 Gb with an average genome sequencing depth of 63X, 89X and 66X for the tumor tissues, adjacent non-tumor tissues and normal blood cells respectively. The percentages of fully called bases for all samples were all above 95%. The percentage of the genomes covered at 10X in the tumor tissues, adjacent tissues and normal blood cells were 98%, 97.4% and 97.3%, respectively. The summary statistics of the sequencing results for each sample are listed in Table 1.

**Table 1:** A summary statistics of whole-genome sequencing results

Sample ID	Gross mapping yield (Gb)	Read Depth	Fully called genome fraction	SNP total count	INS total count	DEL total count	SUB total count	Total CNV segment count
**A357-N**	193.538	66.44	0.971	3492002	295890	299955	95653	246
**A357-TT**	185.055	64.19	0.961	3486488	272954	274526	98051	330
**A357-TA**	189.624	66.12	0.956	3457971	277212	280823	101310	360
**A355-N**	194.519	66.57	0.974	3469773	303858	302061	94328	251
**A355-TA**	184.075	63.32	0.969	3342011	280658	279064	92470	409
**A355-TT**	290.237	100.78	0.96	3475951	305251	300437	107964	362
**A368-N**	187.147	64.78	0.963	3460780	279997	286541	99381	243
**A368-TT**	296.613	103.31	0.957	3473247	303717	291929	111119	390
**A368-TA**	173.431	59.97	0.964	3470317	270147	270543	95311	529

Abbreviations: N = normal blood cells, TT = tumor cells, TA = adjacent non-tumor cells.

### The mutational landscape of matched tumor, non-tumor (somatic) and blood white cell (germline) samples of HCC patients

**Figure 1:**
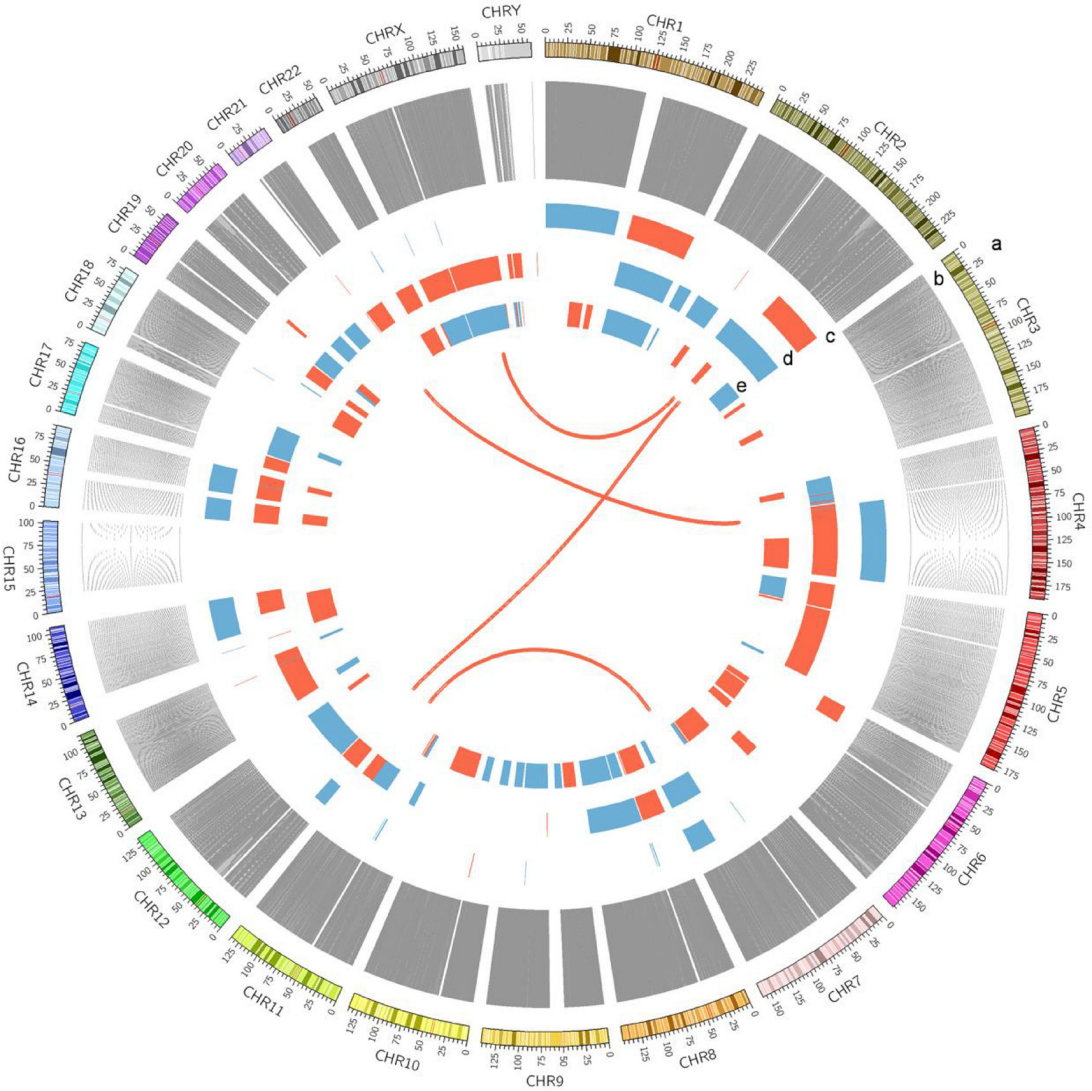
A Circos plot showing the summary of somatic genomic alterations in the three HCC patients Various types of somatic alterations in three HCC tumor genomes were shown in different colored lines or bars. High confidence somatic inter chromosomal SVs are shown as lines in red. Somatic copy number alterations are shown as bar plots with copy number gain in red and loss in blue. Each circus from outer to inner circles represents different kinds of genomic alterations in the study. a, chromosome number. b, line plots of union of somatic SNVs in 3 patients. c, copy number alterations in patient A368. d, copy number alterations in patient A355. e, copy number alterations in patient A357.

Clinical information of the three HCC patients is summarized in Supplementary Table 1. We detected all kinds of somatic alterations including nonsilent somatic mutations, structural variations (SVs) and copy number variations (CNVs) in the three way comparisons of tumors vs. adjacent tissues, tumors vs. germline cells (blood), and adjacent tissues vs. germline cells (blood) (Figure 1). The nonsilent somatic mutations include missense mutations, nonsense single nucleotide variations (SNVs), substitutions, splice-site mutations as well as frameshift indels (insertions and deletions).

We were most interested in variations that are unique to the tumor and/or unique to the adjacent non-tumor tissues compared with the germline cells. By a systematic comparison among tumor mutations in all three samples (union of three), adjacent-tissue mutations in all three samples and normal variations (as the baseline) in all three normal blood samples, we classified the mutations into 3 groups: the tumor unique, the adjacent non-tumor unique and the shared tumor-and-adjacent (alterations that were shared by the tumor and adjacent non-tumor tissues, but absent in the white blood cells).

**Figure 2:**
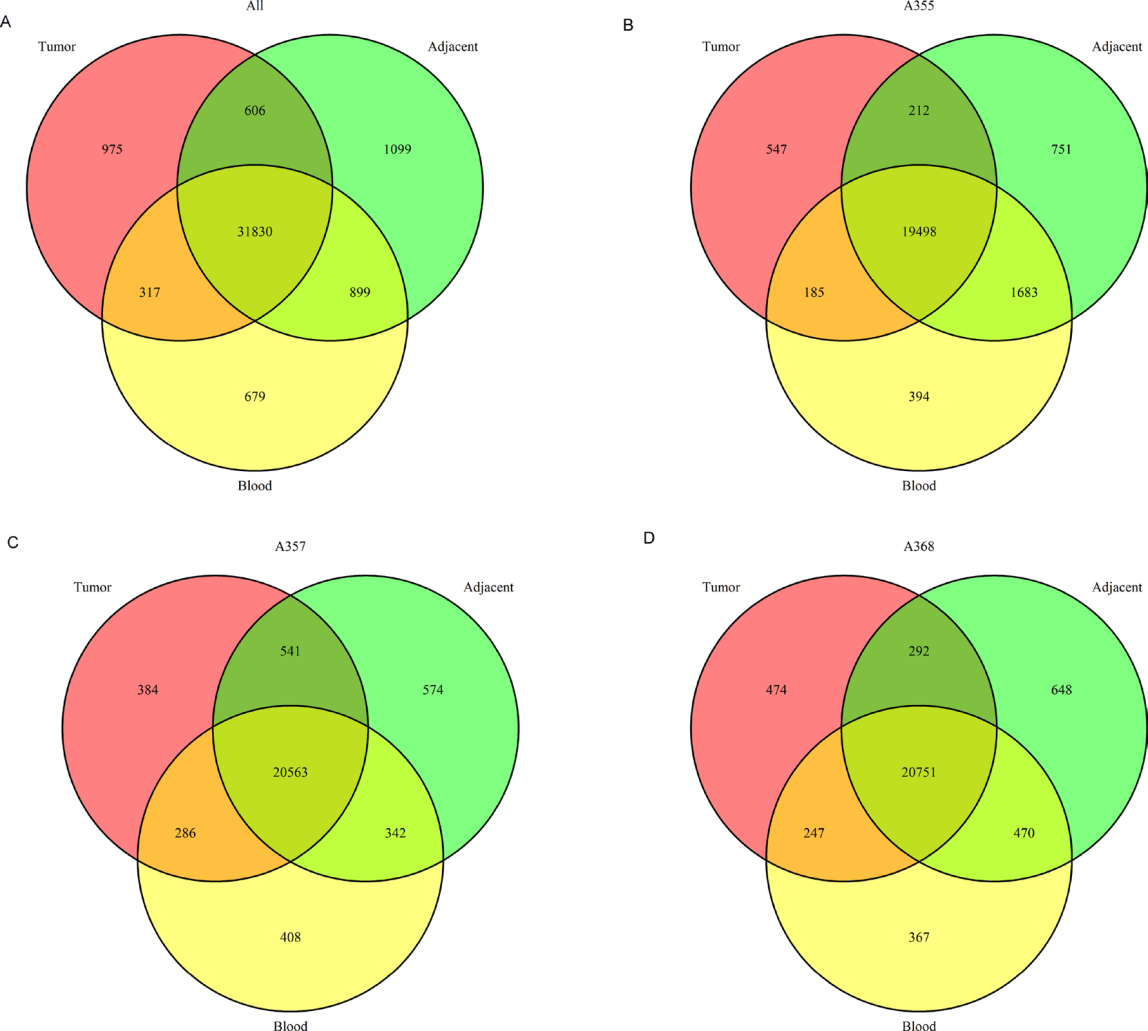
The number of single nucleotide variations throughout the genomes of the tumor and adjacent non-tumor tissues and the matched normal blood cells (**A**) Venn diagram illustrates overlapped SNVs among the union of the tumors, the adjacent tissues and the blood samples. B, C and D show overlapped SNVs for each patient (**B**) patient A355; (**C**) patient A357; (**D**) patient A368, respectively.

We found that the average number of the unique somatic mutations in each tumor sample and adjacent non-tumor tissue is 59,136 and 83, 633, respectively. Among them, only 25, 493 SNVs were shared by the tumor and the adjacent tissues while absent in the white blood sample. A low number of shared SNVs between the tumor and the adjacent non-tumor tissue suggests an evolution theory of positive (Darwinian) selection of advantageous mutations during tumor progression. We hypothesize that the shared mutations between the tumor and the adjacent non-tumor tissues (both were compared with the germline) might be driver mutations for hepatocarcinogenesis while those unique to the adjacent non-tumor mutations might be selected against during tumorigenesis or tumor evolution, and finally, those unique to the tumor tissues might evolve later in the tumor progression. By these systematic analyses, we can draw a picture of the dynamics of mutation profiles during tumor progression. The overlaps of exonic and splice site mutations among the union of three tumors, the union of three adjacent non-tumor tissues and the union of three normal white blood cell samples were shown as a Venn diagram in Figure 2A. In addition, Venn diagrams of overlapped mutations among the tumor, the tumor adjacent tissue and the normal white blood cell sample for each patient were shown in Figure 2B, 2C and 2D respectively (B, patient A355; C, patient A357; D; patient A368).

**Figure 3:**
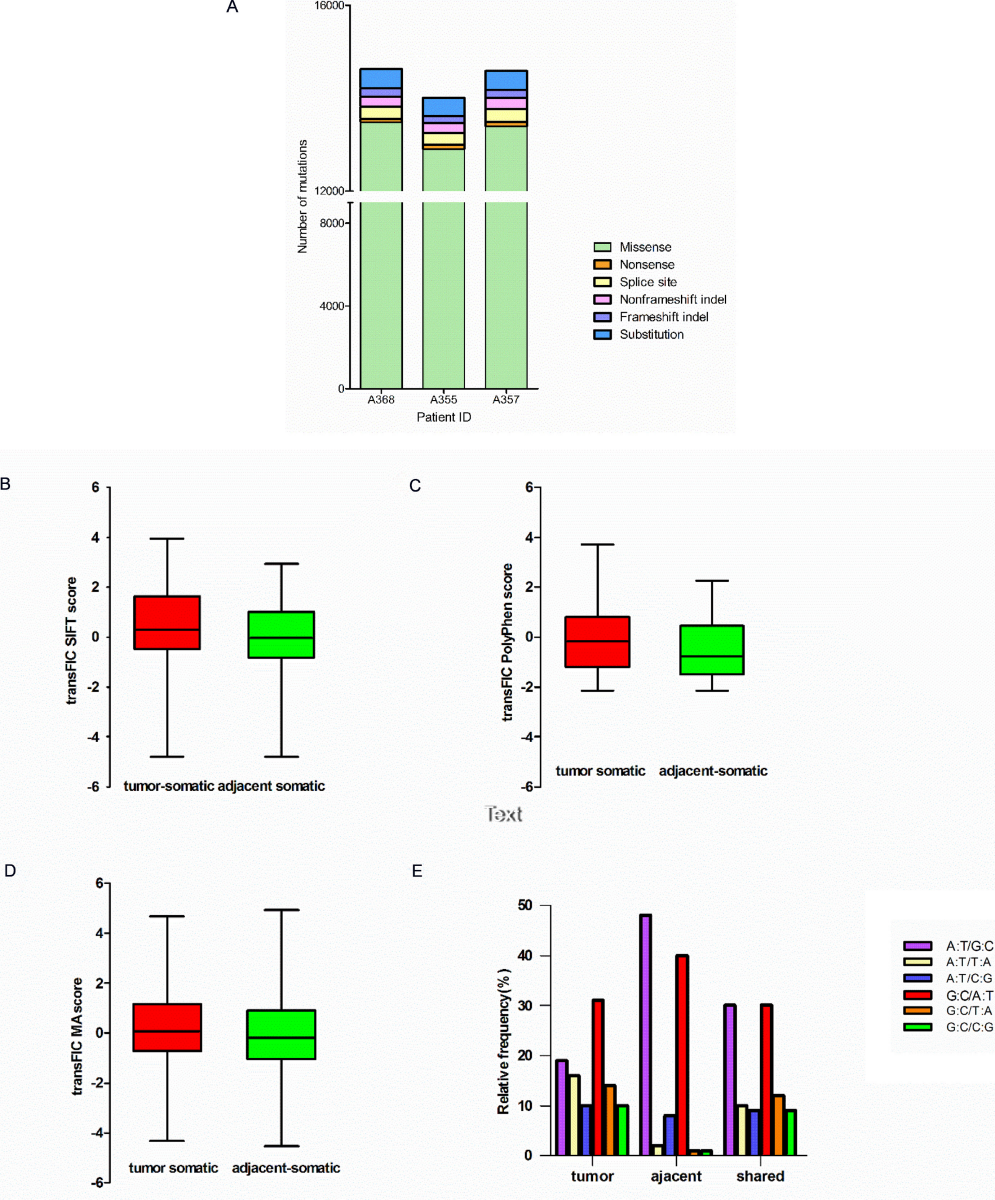
Characteristics of mutations in HCC patients (**A**) Distributiong of nonsilent mutations in tumor of each HCC patient. Bar charts of the scores of SIFT (**B**), Polyphen2 (**C**) and Mutation Assessor (**D**) Of unique nonsynonymous point mutations between tumor and adjacent tissues (all with Mann-Whitney test *P* < 0.001). (**E**) Mutation spectrum of transition and transversion categories in tumor and adjacent tissues.

The exonic and splice site mutations are more important than the mutations in other genomic regions because they usually have significant functional consequences. We therefore focused on the analysis of identifying SNVs occurred in exons and splice sites of the tumors and the adjacent tissues. In the end, we identified 665 nonsilent somatic mutations in the tumors (union of three samples) including 113 frameshift indels, 23 non-frameshift indels, 48 short stretch substitutions, 448 missense and 33 nonsense mutations (Supplementary Table 2). In the adjacent non-tumor tissues (union of three samples), we identified a total 692 nonsilent somatic mutations including 138 frameshift indels, 52 non-frameshift indels, 53 substitutions, 451 missense mutation, and 11 nonsense mutations (Supplementary Table 3). Comparing mutations among the tumors, the adjacent non-tumor tissues and the normal white blood cells, 294 nonsilent mutations were shared by the tumors and the adjacent tissues, but absent in the blood cells. They include 26 frameshift indels, 16 nonframeshift indels, 9 substitutions, 237 missense SNVs and 8 nonsense mutations (Supplementary Table 4). Distribution of nonsilent mutations in the tumor of each HCC patient was shown in Figure 3A. The proportions of each category of nonsilent mutations in the tumors are distributed similarly and the majority of the mutations are missense mutations (Figure 3A).

To predict functional effects of the nonsilent somatic mutations, we used a computational algorithm transFIC (TRANS formed Functional Impact for Cancer), which uses the scores provided by well-known tools (e.g. SIFT, Polyphen2, MutationAssessor) to rank the functional impact of cancer somatic mutations. The transFIC analysis revealed that the scores of SIFT, Polyphen2 and Mutation Assessor prediction of somatic missense mutations in the tumors are higher than the scores in the adjacent tissues (Mann-Whitney test *P* < 0.001) (Figure 3B, 3C and 3D) in all three cases, suggesting that the tumor mutations seem to have more severe functional consequences.

We also determined the mutation spectrum of transition and transversion categories for the HCC patients (Figure 3E). The results showed that the mutation categories shared in both the tumor and the adjacent non-tumor tissues are primarily G C/A·T and A·T/G C transversions. The mutation categories in the tumors consist of more G C/C G, C G/T A and A·T/T A transversions compared with those in the adjacent tissues.

### Validation of mutations by sequenom MassARRAy

We next sought to validate interesting missense and nonsense mutations identified in our study. We picked exonic SNPs of interesting genes such as TP53 and VCX for validation among the list of tumor associated genes with nonsilent mutations in the tumors, the adjacent tissues and the while blood cells (Supplementary Tables 5, 6, 7). We employed an orthogonal and alternative technology Sequenom MassARRAY for validation in a new set of 177 samples from HCC patients.

**Figure 4:**
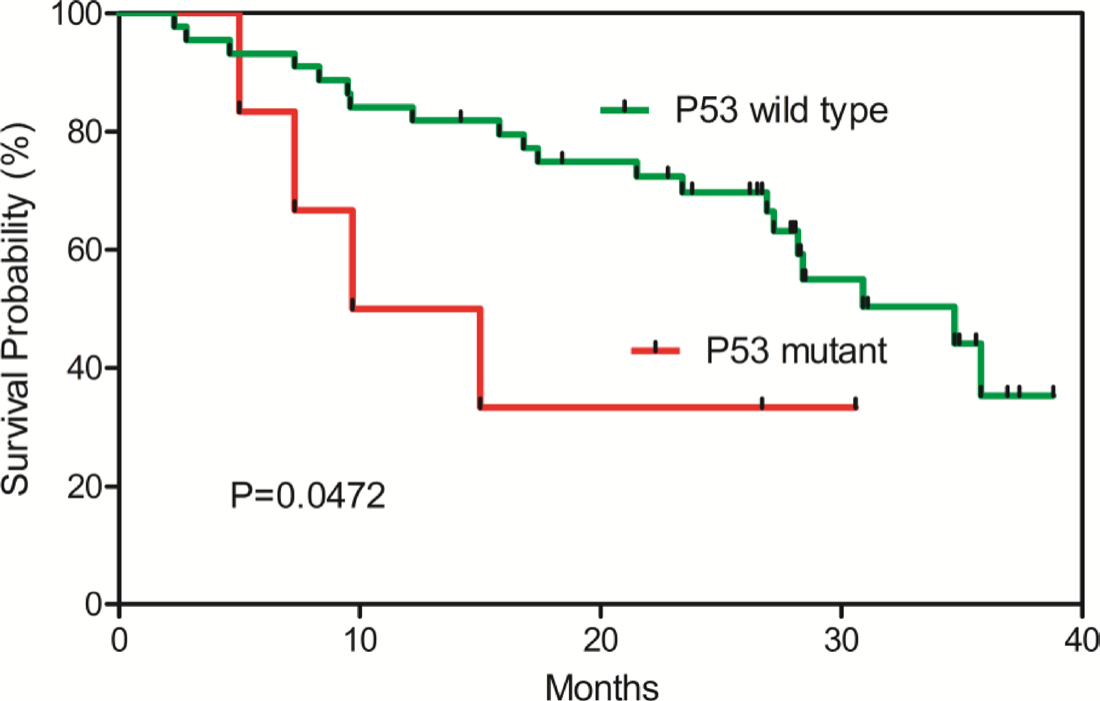
Kaplan-Meier survival plot for TP53 (R249S) wild-type and mutant HCC patients HCC patients with the TP53 (R249S) mutations have significant poor survival.

From the validation analysis, we found that the TP53 (R249S) mutation was found exclusively in the tumor tissues occurring in 7.7% of the HCC patients (Supplementary Table 8). Furthermore, a survival analysis of the HCC patients with or without the TP53 (R249S) mutation showed that the HCC patients with the TP53 R249S mutation have significantly poor survival compared with those with the wild type P53 alleles (Figure 4). In addition, we found that the L104P mutation in the VCX gene (Variable charge, X-linked) was detected with increasing frequencies from the normal, the adjacent tissues to the tumor tissues—frequencies of 14.6% in the HCC tissues, 11.1% in the adjacent tissues, and absent (0%) in the white blood cell samples (Supplementary Table 8), suggesting that this mutation might be a tumor driver gene driving HCC carcinogenesis.

### Analysis of mutated genes with high confidence and significantly enriched pathways

To understand the overall picture of the significant mutations in the tumor and the adjacent tissues, we focused on the list of genes with high-confidence nonsilent somatic mutation in the exons and splice sites identified using the CGI pipeline [24], The lists consist of 558 genes and 560 genes for the tumor and the adjacent tissues respectively (Supplementary Tables 5, 6). Between them, there are 251 shared genes (Supplementary Table 7). We identified 506 genes with high-confidence nonsilent somatic mutations only in the tumor tissues but not in the adjacent tissues. These genes might be better candidates for driver genes (Supplementary Table 5).

To identify additional potential driver genes for HCC carcinogenesis, we further selected genes with recurrent mutations that were predicted to alter their functions and genes with literature-reported roles in carcinogenesis, in particular for HCC. The resulting list contains key cancer-associated and tumor suppressor genes (Tables 2, 3). These genes were mapped to core pathways of chromatin modification (ARID1B, MLL3, MLL2, CREBBP and NCOR1, EP300), transcriptional regulation (GATA3), pathway of APC (AXIN1), STAT (MPL), NOTCH (NOTCH1 and NOTCH2), cell cycle/ apoptosis (TP53, RB1), RAS (CIC, FLT3) and DNA damage control (MSH6).

**Table 2:** The cancer driver and supressor genes found in the tumor tissues of the HCC patients

Gene	Classification	Core pathway	Process	mutation	dbSNP or not	chr	start	end	ref	obs	A355	A357	A368
ARID1B	TSG	Chromatin Modification	Cell Fate	nonsynonymous SNV	novel	6	157150542	157150542	A	T	1	0	0
AXIN1	TSG	APC	Cell Fate	stopgain	novel	16	338223	338223	C	A	1	0	0
				stopgain	novel	16	396701	396701	C	A	0	1	0
CREBBP	TSG	Chromatin Modification; Transcriptional Regulation	Cell Fate	stopgain	novel	16	3778795	3778795	G	A	0	1	0
GATA3	TSG	Transcriptional Regulation	Cell Fate	nonframeshift substitution	novel	10	8097738	8097739	GT	CG	1	0	0
MLL3	TSG	Chromatin Modification	Cell Fate	frameshift insertion	novel	7	151874147	151874147	-	T	0	0	1
				nonsynonymous SNV	yes	7	151919751	151919751	A	G	1	0	0
				stopgain	yes	7	151945071	151945071	-	T	0	0	1
				CNV deletion	novel	7	151900000	152200000			1	0	0
MPL	Oncogene	STAT	Cell Survival	nonsynonymous SNV	novel	1	43803860	43803860	C	A	0	1	0
NOTCH1	TSG	NOTCH	Cell Fate	frameshift insertion	novel	9	139399408	139399408	-	C	0	1	0
NOTCH2	TSG	NOTCH	Cell Fate	nonsynonymous SNV	yes	1	120611960	120611960	C	T	1	1	0
TP53	TSG	Cell Cycle/Apoptosis; DNA Damage Control	Cell Survival	nonsynonymous SNV	yes	17	7577534	7577534	C	A	0	1	0

**Table 3:** The cancer driver and supressor genes found in the adjacent tissues of the HCC patients

Gene	Classification	Core pathway	Process	mutation	dbSNP or not	chr	start	end	ref	obs	A355	A357	A368
ARID1B	TSG	Chromatin Modification	Cell Fate	frameshift deletion	novel	6	157222580	157222580	A	-	1	0	0
CIC	TSG	RAS	Cell Survival	nonframeshift substitution	novel	19	42796589	42796590	GC+A12:I21	AG	0	0	1
EP300	TSG	Chromatin Modification; APC; TGF-β; NOTCH	Cell Survival/Fate	nonframeshift substitution	novel	22	41545942	41545945	CAGC	GCAA	0	1	0
FLT3	Oncogene	RAS; PI3K; STAT	Cell Survival	nonsynonymous SNV	yes	13	28674628	28674628	T	C	0	1	0
MSH6	TSG	DNA Damage Control	Genome Maintenance	nonframeshift substitution	novel	2	48028228	48028229	CT	AG	0	0	1
NCOR1	TSG	Chromatin Modification	Cell Fate	stopgain	novel	17	15983770	15983770	G	A	0	0	1
RB1	TSG	Cell Cycle/Apoptosis	Cell Survival	nonsynonymous SNV	novel	13	49051518	49051518	T	G	1	0	0
MLL3	TSG	Chromatin Modification	Cell Fate	nonsynonymous SNV	yes	7	151919690	151919690	C	T	0	0	1
MLL2	TSG	Chromatin Modification	Cell Fate	nonsynonymous SNV	novel	12	49427278	49427278	T	G	1	0	0

Gene Ontology analysis revealed that the mutated genes in the tumors were significantly enriched in the categories of extracellular matrix (by cellular component) and extracellular matrix structural constituent (by molecular function) (Supplementary Figure 1). The mutated genes in the adjacent tissues were enriched in the categories of intermediate filament cytoskeleton, intermediate filament and keratin filament (Supplementary Figure 1). We further performed an impact analysis for over-represented pathways using a web-based Pathway-Express [25] (http://vortex.cs.wayne.edu/ontoexpress/), and identified 12 pathways and 14 pathways that were significantly enriched at Bonferroni corrected *P* value level of < 0.05 in the tumor tissues and the adjacent tissues respectively (Table 4). Notably, tumor extracellular matrix (ECM) receptor interaction and cell adhesion molecules (CAMs) were the top two most significantly enriched pathways in the tumor tissues (*P* value 7.54E-10 and 2.41E-09). In the adjacent tissues, olfactory transduction was the most significantly enriched pathway with *P* value of 8.49 E-12. There are 11 pathways that are shared between the tumor and adjacent tissues (Table 4), which include the neuroactive ligand-receptor interaction, the ABC transporters, the calcium signaling pathway, the phosphatidylinositol signaling system, the GnRH signaling, the hematopoietic cell lineage, the focal adhesion and the gap junction pathways.

**Table 4:** Pathway impact factor analysis for significantly (significance at the Bonferroni corrected *P* value level of 5%) enriched KEGG pathways

Comparison	Pathway Name	Impact Factor	%Pathway Genes in Input	Corrected *p*-value	Classified Group
Tumor unique	ECM-receptor interaction	25.54	90.48	7.54E-10	Tumor environmental information processing
	Cell adhesion molecules (CAMs)	24.38	78.36	2.41E-09	
Adjacent non-tumor unique	Olfactory transduction	25.50	78.27	7.90E-10	Organismal systems
Intersection of tumor and adjacent non-tumor	Neuroactive ligand-receptor interaction	20.19	66.41	1.58E-07	Tumor environmental information processing
	ABC transporters	12.47	81.82	3.59E-04	
	Calcium signaling pathway	14.89	66.48	3.17E-05	
	Phosphatidylinositol signaling system	14.69	76.32	3.88E-05	
	Complement and coagulation cascades	12.65	75.36	2.98E-04	Organismal systems
	Taste transduction	8.92	73.59	0.0125	
	GnRH signaling pathway	8.70	66.02	0.015	
	Hematopoietic cell lineage	8.01	66.67	0.031	
	Gap junction	9.522	67.71	0.0068	Cellular process
	Focal adhesion	16.46	66.50	6.63E-06	
	Pathways in cancer	8.61	57.88	0.017	Human disease

### Structure variations detection and validation

**Figure 5:**
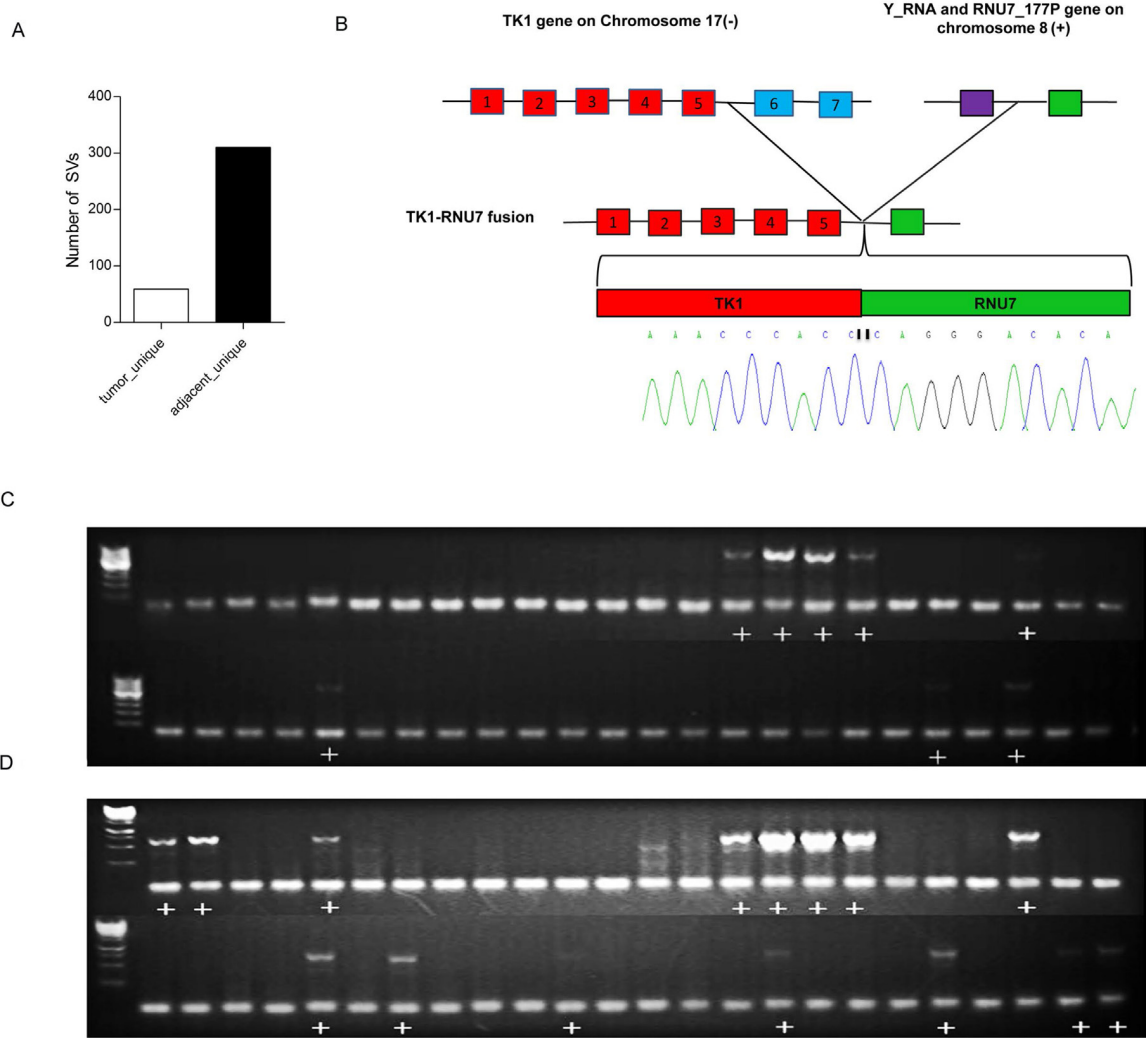
Structural variations of HCCs (**A**) The number of unique structure variations (SV) predicted in the tumors and the adjacent non-tumor tissues. The number of SVs in the adjacent tissues is more than 5 times the SVs in the tumor tissues. (**B**) Sequencing validation of the PCR product harboring the TK1-RNU7 fusion. The TK1 gene on chromosome 17 was broken after the fifth exon and fused with the upstream regulating region of the RNA gene RNU7-177P on chromosome 8. The breakpoint was amplified by PCR from the tumor tissues and subjected to Sanger Sequencing to verify the fusion. The sequencing chromatographs covering the breakpoint are shown. Red box=exons, purple box=Y-RNA gene, green box=RNU7-177P gene. (**C**) PCR analysis of TK1-RNU7 fusion in a cohort of 48 tumors. (**D**). PCR analysis of TK1-RNU7 fusion in the cohort of matched 48 adjacent tissues. The PCR product with the fusion is 556bp. The figure shows fusion was detected both in tumors and adjacent tissues, but more in adjacent. Plus sign (+) means fusion was detected.

Recurrent genomic rearrangements are characteristic features of many human cancers [26–29]. We identified a large number of genomic rearrangements including large intra-chromosomal deletions, inversions and inter-chromosomal fusions (Supplementary Tables 9, 10). Interestingly, the number of unique intra-chromosomal somatic structural variations in the adjacent non-tumor tissues was more than 5-fold higher than that in the tumor tissues (Figure 5A). However, we identified five unique inter-chromosomal SVs that occurred in the tumors but not in the adjacent non-tumor tissues. We found a TK1-RNU7 fusion between the TK1 gene in chr.17 and the genomic region located between the miscRNA Y-RNA gene and the snRNA RNU7-177P gene in chromosome 8, which would result in a truncated thymidine Kinase1 (TK1). We then designed a PCR amplification and sequence strategy to verify this fusion event and we were able to verify this fusion event at the sequence level (Figure 5B).

We next conducted a PCR validation of the TK1-RNU7 fusion in a cohort of 48 pairs of tumors and adjacent tissues (Figure 5C–5D). The fusion was detected in both the HCC and matched adjacent tissues in 7 patients, and detected only in the tumor tissue in one patient and only in the adjacent tissues in 7 patients. The frequency of this fusion event was calculated as 16.7% (8/48) and 29.2% (14/48) in the tumor and the adjacent tissues respectively, showing a decreasing frequency from the adjacent tissues to the HCC tissues.

### Copy number variations

For CNV analysis, we simply compared the tumor tissues with their corresponding adjacent non-tumor tissues, and identified a number of gained and deleted regions. The gained chromosomal bands include Xp22.33, 19p13.2, 21q22.3 and the deleted bands include 9p11.2, 7q34, 9p21.11, 9q13, 8q24.3, 7q36.2 and 10q11.22. (Supplementary Figure 2, Supplementary Tables 11, 12). The CNVs patterns we found here are similar to what has been reported [30]. Annotation of the genes involved in the CNVs revealed that the 88 copy number gained regions harbored 8333 genes and the 130 copy number deleted regions harbored 9771 genes. Pathway analysis of these altered genes using the KOBAS 2.0 function annotation tool showed 10 HCC related KEGG pathways significantly enriched by the gained genes or the deleted genes (Supplementary Tables 13, 14) (*P* value < 0.05) including the JAK-STAT signaling pathway, whose aberrant activation was reported to contribute to liver cancer [31, 32]. Notably, large copy number loss or gain of ZNFs (zinc finger family) and NBPFs (neurobalstoma breakpoint family) were found in all 3 patients. The copy number alteration of ZNF 692 was found in all of the three tumors. This gene was identified as one of the copy number variation-driven genes for liver cancer previously [33].

## DISCUSSION

The strength of our analysis is the inclusion of normal white blood cell sample in whole genome sequencing of cancers. The three way comparisons of whole genome sequencing data from tumor tissues, adjacent non-tumor tissues and normal white blood cells allow one to get a picture of dynamic changes during tumor progression and to identify those mutations occurring only in tumors and those only occurring in adjacent non-tumor tissues, or with increasing frequencies or decreasing frequencies from adjacent tissues to tumor tissues. Those occurring only in adjacent tissues might be transit mutation or passenger mutations and lost by nature selection during tumor evolution and those occurring only in tumors might confer growth advantage and positively selected (Darwinian selection) and thus might be tumor driver genes. We identified 506 genes with high-confidence nonsilent somatic mutation only in the tumor tissues but not in the adjacent tissues. These genes might be potential candidates for driver-mutation genes (Supplementary Table 5). Our data will contribute to the research community for meta-analysis or additional comparative analysis.

We found that the VCX gene (Variable charge, X-linked) L104P was detected in 14.6% of the HCC tissues and in 11.1% of the adjacent tissues but not in the blood samples. Increasing frequency of the mutation in the VCX gene from zero percent in the blood samples, to 11.1% in the adjacent tissues and then to 14.6% in the tumor tissues suggesting that this mutation might be a tumor driver gene driving HCC carcinogenesis. The VCX gene encodes a small and highly charged protein of unknown function that belongs to the VCX/Y gene family. Previously, Zhang et al. found that the VCX gene was significantly mutated in a large cohort of 231 patients with resectable hepatocellular carcinoma [34].

We found that the TP53 (R249S) mutation was found exclusively in the tumor tissues occurring in 7.7% of the HCC patients. This mutation was further analyzed in a new set of 177 samples from HCC patients with survival data. Interestingly, a survival analysis of the HCC patients with or without the TP53 (R249S) mutation showed that the HCC patients with the TP53 R249S mutation have significantly poor survival (Figure 4). This mutation might be developed into clinical test for monitoring HCC prognosis.

TP53 mutations were implicated in HCC and hotspots of TP53 mutations have been identified as independent predictor of HCC survival [35]. More than 30% HCC patients in the Catalog of Somatic Mutations in Cancer (COSMIC) database have a mutation in TP53, ranking it the most frequently mutated gene for HCC. The R249S mutation resides in a hotspot associated with exposure to aflatoxin B1 (AFB1) in China [36]. We found that TP53 mutations were detected in 10.1% of HCC patients in the validation cohort, suggesting that aflatoxin B exposure could be an etiological factor for these patients. Although the TP53 R249S mutation was not detected in the tumor adjacent tissues, we did identified a frameshift insertion of TP53 shared by the tumor and the adjacent tissues (Supplementary Table 4), suggesting that different mutations in the TP53 genes might contribute differently at different stages of HCC development.

We identified and validated a TK1-RNU7 fusion (Figure 5) between the TK1 gene in chr.17 and the genomic region located between the miscRNA Y-RNA gene and the snRNA RNU7-177P gene in chromosome 8, which would result in a truncated thymidine Kinase1 (TK1). Several studies have shown that TK1 is involved in DNA repair and up-regulated during S phase [37]. TK1 activity is very low or absent in nondividing cells but increases during G1/S phase, and then disappears rapidly in G2/M phase [38]. The C-terminal end of TK1 harbors a specific KEN box motif recognized by the ubiquitin-dependent proteasome for proteolytic degradation of TK1 [38]. It was demonstrated that a deletion of the 40 C-terminal amino acids of TK1 delayed the G2/M phase specific degradation [39]. The TK1-RNU7 fusion identified herein would result in a deletion of 103 amino acids from its C-terminal. The frequencies of this fusion event decreased from the adjacent tissues (29.2%) to the tumors (16.7%), suggesting that a truncated thymidine Kinase1 (TK1) caused by the fusion event might be deleterious and be selected against during tumor progression. Future investigation of the function consequences of this fusion event is warranted.

Pathway analysis revealed that the tumor extracellular matrix (ECM) receptor interaction and the cell adhesion molecules (CAMs) pathway were the top two most significantly enriched pathways in the tumor tissues (*P* value 7.54E-10 and 2.41E-09). In the hepatic microenvironment, ECM provides biochemical and mechanical cues to the tumor cells and surrounding tumor microenvironment. The components of ECM, such as collagens, laminins, fibronectin, glycosaminoglycan and proteoglycans, interact directly and indirectly with HCC cells, leading to both the changes of phenotype and function of HCC [40]. We found 17 collagens, 20 integrins and twelve laminins were mutated in HCC. ECM-receptor interaction was also reported to be associated with the venous metastases of HCC with PVTT (Portal vein tumor thrombus) [41]. We also identified three other pathways involved in tumor microenvironment that are significantly enriched in the mutated genes, including the ABC transporters, the calcium signaling transduction and the neuroactive ligand-receptor interaction pathways. Mutations in the ABC transporters pathway were shown to be associated with better differentiation of cells [41]. For the calcium signaling transduction, increases in intracellular Ca2+ concentration either in space or time or amplitude have been shown to be important in cell migration [42].

## CONCLUSIONS

The three-way comparisons of normal blood white cells, adjacent normal tissues and tumor tissues allow the identification of potential driver mutations of carcinogenesis. From such analysis, the TP53 (R249S) mutation was found exclusively in the tumor tissues and the L104P mutation in the VCX gene (Variable charge, X-linked) was detected with increasing frequencies from the normal, the adjacent tissues to the tumor tissues, suggesting that these two mutations might be driver mutations driving HCC carcinogenesis. Indeed, a survival analysis of the HCC patients with or without the TP53 (R249S) mutation showed that the HCC patients with the TP53 R249S mutation have significantly poor survival compared with those with the wild type P53 alleles. Finally, we identified a TK1-RNU7 fusion, which would result in a deletion of 103 amino acids from its C-terminal. The frequencies of this fusion event decreased from the adjacent tissues (29.2%) to tumors the (16.7%), suggesting that a truncated thymidine Kinase1 (TK1) caused by the fusion event might be deleterious and be selected against during tumor progression. In summary, our dataset provides the research community a valuable dataset for identifying dynamic changes of mutation profiles and driver mutations for HCC.

## MATERIALS AND METHODS

### Tumor specimens and ethical statement

This investigation has been conducted in accordance with the ethical standards and according to the Declaration of Helsinki and according to national and international guidelines and has been approved by the authors’ institutional review board. Snap-frozen fresh tumor tissues, adjacent non-tumor liver tissues and blood samples of 3 hepatocellular carcinomas (HCCs) were collected from the Cell Engineering Research Center at The Fourth Military Medical University, Xi'an, China. To assess the prevalence of candidate mutations and structural variations in HCC, an additional 50 pairs of HCC tumor samples and matched non-tumor tissues and also 10 normal blood samples were randomly selected from HBV-related HCC patients in the same center as above. Approval from the institutional review aboard was obtained for this study. Liver tumor specimens were collected at the time of metastasectomy. All frozen sections of HCCs and matched non-neoplastic liver tissues from the 3 patients were reviewed by one professional pathologist. All tumors were microdissected to guarantee > 90% purity of neoplastic cells. Microdissection was performed with a sterile needle stained with nuclear fast red as previously described [43]. All patients did not receive any anti-cancer treatment before surgery. Patient demographics were described in Supplementary Table 1, including age, tumor size, TNM stage, pathologic grade, serum alpha-fetoprotein (AFP) level, portal vein tumor thrombus (PVTT) and Child-Pugh classification.

### DNA preparation, whole genome sequencing and data processing

High-molecular-weight genomic DNAs were extracted from three pairs of fresh frozen tumor tissues, adjacent non-neoplastic specimens and blood samples using the DNeasy Blood and Tissue kit (QIAGEN) according to the manufacturer's recommendations. Briefly, 5–10 μg genomic DNA was used for library generation. Whole genome paired-end sequencing of the matched tumor, adjacent non-tumor and blood samples was performed by Complete Genomics, Inc. (CGI; Mountain View, CA) using unchained combinatorial probe anchor ligation chemistry on arrays of self-assembling DNA nanoballs, as described previously [24]. Paired-end reads were aligned to the NCBI reference genome (Build 37), local *de novo* assembly and variant-calling were performed to identify sequence variation in each sample as previously described [24]. A reference score was calculated for each called base in the genomes [24]. CGA tools v1.3.0 was applied for selecting unique SVs of the three pairwise (http://cgatools.sourceforge.net/docs/1.3.0) and SV annotation. Furthermore, we used custom designed PERL processing routines to perform additional downstream bioinformatics analyses.

### Somatic point mutations, short indel calls and validation

Unique somatic single nucleotide variants including SNPs, indels and substitutions in the following three groups: the tumor unique, the adjacent unique and the shared (alteration is shared by the tumor and the adjacent tissues, but absent in the blood samples) were uncovered using the calldiff function of cgatools (http://cgatools.sourceforge.net/). Next in order to annotate somatic variants, Annovar (http://annovar.openbioinformatics.org/en/latest/) was used to interpret variants and their transcript effects and to determine the presence in dbSNP138 (http://www.openbioinformatics.org/annovar/download/hg19_snp138.txt.gz). TransFIC, a method to transform functional impact scores for cancer (available online at http://bg.upf.edu/transfic/home) was applied to identify somatic nonsynonymous mutations altered exclusively in the tumor tissues to predict putative drivers of tumorigenesis [35]. This transformation allows us to use SIFT, PolyPhen-2 and Mutation Assessor to rank the functional impact of cancer somatic mutations. Mutations with greater transFIC are considered candidate cancer drivers. PROVEAN (Protein Variation Effect Analyzer) was used to predict whether an amino acid substitution caused by short indels has an impact on the biological function of a protein [44]. The performance of PROVEAN is comparable to SIFT or PolyPhen2. Besides, mutations present in the 1000 genome project were excluded [45]. Tumor specific missense and nonsense mutations with high mutation score were validated by Sequenom MassARRAy platform of BGI (BGI, Shenzhen, China) with standard protocols.

### Structural variations (SV) validation

The junctiondiff function of cgatools was used to call the unique somatic structural variations in tumors and adjacent tissues. High confidence SVs were those that had at least 10 matepairs in a cluster, in which *de novo* assembly of the junction was successful, had a high mapping diversity and for which there was an absence of specific repeat sequences on the left and right side of the junction, as previous reported [46]. In order to validate the predicted somatic structural variations with important roles in cancers, PCR was used to amplify TK1-N/A fusion breakpoint junction and then sequenced in another cohort of 48 pairs HCC samples and matched adjacent non-tumor samples with specific PCR primers: sense: 5′ GAACCAAGAGCCATCCCTACCAT 3′; antisense: 5′ CTTTCCAGTTCCCTGACATCGTG 3′. PCR amplification was performed using 2xTaq PCR StarMix (GenStar Biosolutions) with the following steps: step 1: 94°C for 2 minutes; step 2: 35°C cycles of 94°C for 30 s, 60°C for 30 s and 72°C 18 s; step 3: 72°C for 5 minutes followed by conventional Sanger sequencing. Consequently, the sequences were mapped to the reference genome to confirm the breakpoints at single nucleotide resolution.

### Copy number variations

The DepthOfCoverage (https://software.broadinstitute.org/gatk/) analysis was conducted to detect copy number variations (CNVs) in the regions of the genome rich in segmental duplications. Sequence coverages were averaged and corrected for GC bias and normalized for average haploid genome coverage. In the case of comparing the tumor, the adjacent and the normal blood samples, the coverage in the tumor genome is normalized to the coverage for the same region in the matched genome. Besides, a hidden Markov model (HMM) was used to classify copy number values for genome segments

### Functional enrichment analysis

We applied the web-based onto-tool Pathway-Express (http://vortex.cs.wayne.edu/ontoexpress/) to perform impact analysis for over-represented pathways of KEGG of the highly mutated exonic genes in the union of the gene sets of the 3 tumors and that of the 3 adjacent tissues. Besides, Bonferroni correction (BC) correction (significance level of 0.05) was used for identifying significantly affected pathways.

### Statistical analyses

Data analysis was done with GraphPad Prism version 5.0 for Windows (GraphPad Software, San Diego CA). All statistical analysis was performed using SPSS 17.0 software (SPSS Inc., Chicago, IL). For statistical comparison, Student's *t* test, or Mann-Whitney *U* test was performed appropriately. A Circos program, made with the R program, was used to display the genomic alterations in each sample. *P* values less than 0.05 were considered statistically significant.

### Data access

The SNP data from the WGS analysis have been submitted with the data access address: http://www.ncbi.nlm.nih.gov/SNP/snp_viewTable.cgi?handle=SYSTEMSBIOZJU.

## SUPPLEMENTARY MATERIALS FIGURES AND TABLES



























## Abbreviations

WGS: whole genome sequence
HCC: hepatocellular carcinoma
SNV: single nucleotide variation
SNP: single nucleotide polymorphism
Indel: insertion or deletion
SV: structural variation
CNV: copy number variation
HBV: hepatitis B virus
