# Supplementary material for: Whole genome sequencing of matched tumor, adjacent non-tumor tissues and corresponding normal blood samples of hepatocellular carcinoma patients revealed dynamic changes of the mutations profiles during hepatocarcinogenesis
Source: Oncotarget. 2017 Feb 17;8(16):26185–99. doi: 10.18632/oncotarget.15428 (PMC5432249; doi:10.18632/oncotarget.15428)
Supplement: Supplementary file 1 [file oncotarget-08-26185-s001.pdf]

Whole genome sequencing of matched tumor, adjacent non-tumor tissues and corresponding normal blood samples of hepatocellular carcinoma patients revealed dynamic changes of the mutations profiles during hepatocarcinogenesis

Supplementary Materials

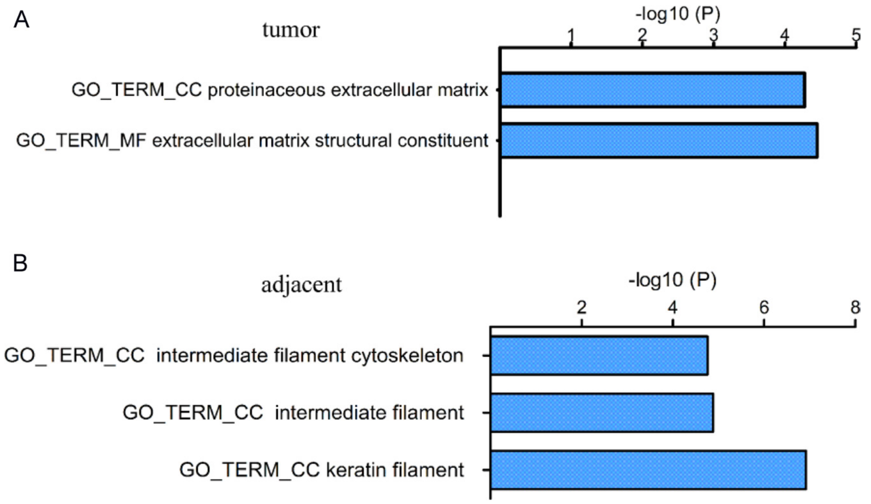

Supplementary Figure 1: Significantly enriched gene ontology (GO) terms of the 572 somatic-nonsilent-mutation-related genes in the tumor tissues and those of the 584 such genes in the adjacent tissues. Adjusted *P* value < 0.05 and False Discover Rate < 0.1.

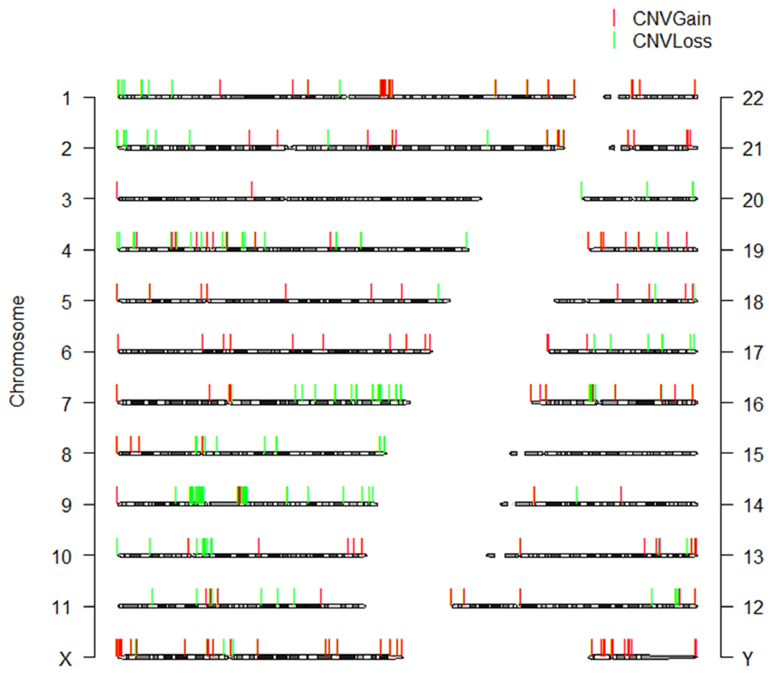

Supplementary Figure 2: The copy number variation (CNV) landscape of the HCC tissues. The CNV data from the three HCC patients were combined. The red color indicates amplification of and the green color indicates deletion of genomic regions.

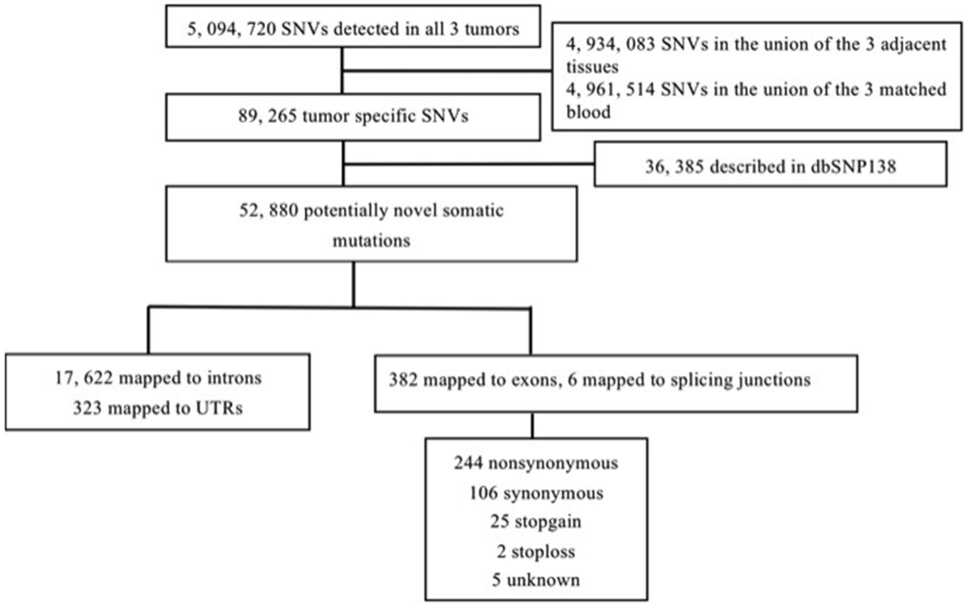

Supplementary Figure 3: A flow chart showing the process of the identification of somatic point mutations including the filtering criteria and the results.

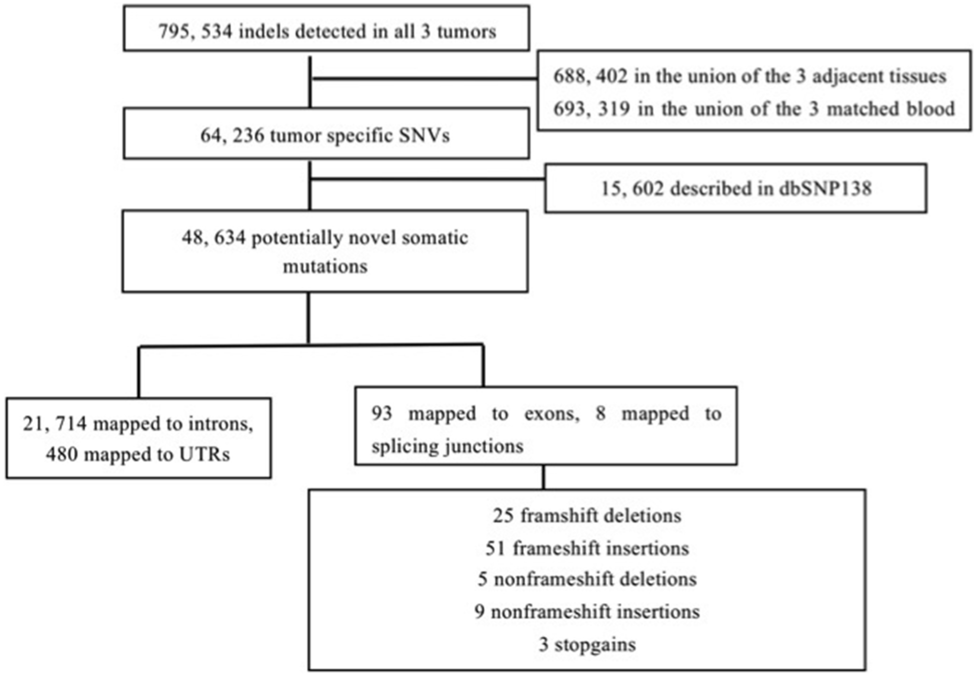

Supplementary Figure 4: A flow chart showing the process of the identification of somatic indels including the filtering criteria and the results.

Supplementary Table 1: Clinical information of the 3 HCC patients. See_Supplemenbtary_Table 1

Supplementary Table 2: List of tumor somatic nonsynonymous SNVs with transFIC score and Indels with PROEVAN score List of tumor somatic nonsynonymous SNVs with transFIC score. See_Supplemenbtary_Table 2

Supplementary Table 3: List of adjacent unique nonsynonymous SNVs with transFIC score and Indels with PROEVAN score Adjacent unique nonsynonymous SNVs with transFIC score. See_Supplemenbtary_Table 3

Supplementary Table 4: List of intersection of nonsynonymous SNVs and Indels between tumor and adjacent tissue but absent in normal blood sample. See_Supplemenbtary_Table 4

Supplementary Table 5: List of tumor associated genes with somatic nonsilent mutation identified in our analysis. See_Supplemenbtary_Table 5

Supplementary Table 6: List of somatic nonsilent mutation related adjacent non-tumor genes. See_Supplemenbtary_Table 6

Supplementary Table 7: List of somatic genes shared by tumor and adjacent non-tumor tissues. See_Supplemenbtary_Table 7

Supplementary Table 8: List of TP53(R249S) and VCX (L104P) mutation in the validation cohort of 177 samples from HCC patients. See_Supplemenbtary_Table 8

Supplementary Table 9: List of somatic SVs in HCC. See_Supplemenbtary_Table 9

Supplementary Table 10: List of unique SVs in adjacent non-tumor tissues of HCC. See_Supplemenbtary_Table 10

Supplementary Table 11: Gained chromosome bands of HCC tumor compared to adjacent non-tumor tissue. See_Supplemenbtary_Table 11

Supplementary Table 12: Deleted bands of HCC tumor compared to adjacent non-tumor tissue. See_Supplemenbtary_Table 12

Supplementary Table 13: Enriched KEGG pathways in CNV gained genes

#Term	Database	ID	Input number	Background number	*P*-Value	Corrected *P*-Value
Alcoholism	KEGG PATHWAY	hsa05034	73	180	4.69E-7	0.000
Systemic lupus erythematosus	KEGG PATHWAY	hsa05322	55	132	6.15E-6	0.001
Hepatitis C	KEGG PATHWAY	hsa05160	47	135	0.001	0.077
Jak-STAT signaling pathway	KEGG PATHWAY	hsa04630	52	160	0.002	0.102
Cytosolic DNA-sensing pathway	KEGG PATHWAY	hsa04623	26	63	0.002	0.102
Regulation of autophagy	KEGG PATHWAY	hsa04140	19	40	0.002	0.102
Toll-like receptor signaling pathway	KEGG PATHWAY	hsa04620	37	108	0.003	0.140
RIG-I-like receptor signaling pathway	KEGG PATHWAY	hsa04622	26	70	0.005	0.202
Glutathione metabolism	KEGG PATHWAY	hsa00480	19	51	0.016	0.477
Viral carcinogenesis	KEGG PATHWAY	hsa05203	57	207	0.016	0.477
Influenza A	KEGG PATHWAY	hsa05164	49	175	0.019	0.519
Measles	KEGG PATHWAY	hsa05162	40	138	0.021	0.526
Cytokine-cytokine receptor interaction	KEGG PATHWAY	hsa04060	68	265	0.030	0.590
Parkinson's disease	KEGG PATHWAY	hsa05012	38	134	0.031	0.590
Natural killer cell mediated cytotoxicity	KEGG PATHWAY	hsa04650	37	130	0.032	0.590
Arachidonic acid metabolism	KEGG PATHWAY	hsa00590	21	64	0.032	0.590
Autoimmune thyroid disease	KEGG PATHWAY	hsa05320	17	51	0.045	0.786

Databases: KEGG PATHWAY Statistical test method: hypergeometric test/Fisher's exact test FDR correction method: Benjamini and Hochberg.

Supplementary Table 14: Enriched KEGG pathways in CNV deleted genes

#Term	Database	ID	Input number	Background number	*P*-Value	Corrected *P*-Value
Metabolic pathways	KEGG PATHWAY	hsa01100	323	1240	0.017	0.100
Synaptic vesicle cycle	KEGG PATHWAY	hsa04721	24	64	0.029	0.100
Galactose metabolism	KEGG PATHWAY	hsa00052	14	31	0.030	0.100
Pathogenic Escherichia coli infection	KEGG PATHWAY	hsa05130	21	56	0.039	0.100
Pancreatic secretion	KEGG PATHWAY	hsa04972	32	96	0.043	0.100
Vibrio cholerae infection	KEGG PATHWAY	hsa05110	19	50	0.044	0.100
Endocytosis	KEGG PATHWAY	hsa04144	76	264	0.044	0.100

Databases: KEGG PATHWAY Statistical test method: hypergeometric test/Fisher's exact test FDR correction method: Benjamini and Hochberg.
